# The emerging role and clinical significance of circRNAs in papillary thyroid cancer

**DOI:** 10.3389/fendo.2024.1351776

**Published:** 2024-03-13

**Authors:** Jing Ma, Jing Xu, Xiaoqi Zhang, Jinxing Quan

**Affiliations:** ^1^ The First Clinical Medical School, Lanzhou University, Lanzhou, Gansu, China; ^2^ Department of Endocrinology, Gansu Provincial Hospital, Lanzhou, Gansu, China; ^3^ Department of Endocrinology, Key Laboratory of Endocrine and Metabolic Diseases of Gansu Province, Lanzhou, Gansu, China; ^4^ The Second Clinical Medical School, Lanzhou University, Lanzhou, Gansu, China

**Keywords:** papillary thyroid cancer, circRNA, signaling pathway, biomarker, carcinogenesis

## Abstract

Papillary thyroid cancer (PTC) is the most common type of thyroid malignancy, and its global incidence has been gradually increasing. For advanced PTC, the mortality rates are also increasing yearly. Despite advancements in diagnosis and treatment, some advanced PTC exhibit aggressive behaviors, leading to a poor prognosis. CircRNAs are a class of non-coding RNAs characterized by a covalently closed loop structure. Their stability and abundance have positioned them as promising diagnostic and prognostic biomarkers. Numerous studies have identified dysregulated circRNAs in PTC tissues and cell lines, suggesting their involvement in PTC initiation and progression. In this review, we provide an overview of circRNAs and systematically discuss their role in PTC. CircRNAs affect cancer progression by regulating the Wnt/β-catenin, PI3K/AKT, MAPK pathways, and others. Furthermore, circRNAs have been implicated in PTC metastasis and chemoresistance. We highlight their potential value as diagnostic markers, therapeutic targets, and prognostic indicators. In conclusion, circRNAs play a critical role in PTC, and dysregulated circRNAs influence multiple signaling pathways and cellular processes involved in tumorigenesis and metastasis. It represents a promising avenue for advancing the diagnosis, management, and treatment of PTC.

## 1 Introduction

Thyroid cancer (TC) is the most common endocrine malignancy and accounts for nearly one-third of all malignant tumors of the head and neck. For individuals with advanced TC, new systemic treatments and management are being investigated (1). The global prevalence of TC has been gradually increasing, particularly among young adults and teenagers. The age-standardized incidence of TC is approximately three times higher in women (10.1 per 100,000) than in men (3.1 per 100,000) (2). According to the latest cancer statistics reports in 2022, the number of TC survivors has jumped to the third highest rate in women, and the high-risk age range is 30-45 years old (3). Based on the Adolescents and Young Adults (AYAs) 2020 statistics, the 15-29 age group has the highest incidence of TC (4). Furthermore, TC incidence rates also show significant differences across settings, such as more than a 15-fold difference in incidence rates among women in different regions of the world (2). There are three main histological types of TC, differentiated thyroid cancer (DTC), anaplastic thyroid cancer (ATC), and medullary thyroid cancer (MTC). DTC includes papillary thyroid cancer (PTC) and follicular thyroid cancer (FTC). PTC is the most common type of TC, accounting for nearly 85% of cases. Some aggressive PTC with local or distant metastasis, structural recurrence, and even progression to high-grade cancer can be life-threatening (5). The 5-year survival rate for patients with advanced PTC is only 59% (6). Recently, WHO has made significant adjustments to the classification of TC, highlighting differences in cellular origin, pathological features, molecular classification, and biological behavior into benign, low-risk, and malignant tumors, while also emphasizing the role of biomarkers in aiding diagnosis and determining prognosis (7).

Currently, sampling via ultrasound-guided fine-needle aspiration (FNA) is a commonly used diagnostic way for evaluating malignant thyroid nodules (8). Studies have shown that FNA reduces unnecessary thyroid surgery by 25%. Nonetheless, approximately 30% of thyroid nodules remain undiagnosed by pathology (6, 9), which even leads to misdiagnosis (9). In addition, the diagnostic value of FNA is relatively low for FTC (10). Unnecessary FNA can also add to the burden on the health care system and cause considerable anxiety for patients (11). Therefore, greater focus on accurate diagnosis, management, and treatment of TC is imperative. Understanding the underlying molecular mechanisms of TC can aid in the development of targeted and specialized treatments.

Circular RNAs (circRNAs) are a new class of non-coding RNA molecules and have been found to be widely expressed in various human cancers. Recently, they have emerged as a hot topic due to their potential role in carcinogenesis and tumor progression (12). Unlike linear RNA molecules, circRNAs are generated by head-to-tail splicing and form a closed-loop structure without 5’ to 3’ polarity and a polyadenylated tail. They are highly stable and resistant to degradation and play important regulatory roles in gene expression and other cellular processes (13–15). The study of circRNAs in PTC has been growing rapidly, and recent findings have shed light on their contribution to the development and progression of this disease (12–14). In this review, we give a brief description of circRNAs, then systematically discuss the role of circRNAs in PTC and their potential value in diagnosis, prognosis, and treatment.

## 2 Circular RNAs

CircRNAs were first discovered in the 1970s but were initially thought to be rare and non-functional byproducts of RNA splicing. It was not until the advent of high-throughput sequencing technologies in the early 2010s that circRNAs were found to be widespread and abundant in eukaryotic cells, including human cells. It has undergone rapid development in the past decade and has opened up new avenues for research into gene regulation, biological processes, and cellular signaling. They are mainly categorized according to their genomic origin and the way they are generated. Exonic circRNAs (ecircRNAs) are mainly derived from single or multiple exons, representing the majority of circRNAs (more than 80%). Intronic circRNAs (ciRNAs) contain only introns and are the most common transposons in the genome. Exon-intron circRNAs (EIciRNAs), which contain both exons and introns. In addition, a novel class of circRNAs, namely tRNA intronic circRNAs (tricRNAs), are formed by pre-tRNA splicing. Currently, most of the identified circRNAs belong to ecircRNA (16).

The massive number of circRNAs identified so far suggests that they may have complicated and diverse roles. To date, the functions of circRNAs that have been reported are diverse and include competing endogenous RNAs (ceRNAs) or miRNA sponges, interacting with RNA-binding proteins (RBPs), regulating the splicing or transcription of genes, translation into proteins or small peptides, epigenetic regulation, and modulating the stability of mRNAs (17). What’s more, studies have reported that circRNAs can function in gene regulation by competing with linear splicing (18). Ho et al. (19) found that heterogeneous nuclear ribonucleoprotein M (HNRNPM) can control circRNA biogenesis and thus promote solid tumor development. The most thorough research has focused on circRNAs acting as miRNA sponges. CDR1as, also known as ciRS-7, harbors more than 70 conserved binding sites, is highly expressed in human and mouse brains and was reported to function as a sponge for miR-7 (20). In addition, circITCH, a recently discovered circRNA, similarly acts as a miRNA sponge via miR-7 to promote osteosarcoma migration and invasion (21). All these findings above indicate that circRNAs could function as miRNA sponges to contribute to the regulation of cancer. For the RBPs mechanism, it has been shown that EWS RNA-binding protein 1 (EWSR1) promotes circNEIL3 biogenesis in gliomas (22). Research found that NOVA2 can act as a neural-enriched RBP to promote circRNA biogenesis in the mouse brain (23).

There is also high tumor specificity in the peptide encoded by circRNAs. For example, Zhang et al. found that SHPRH-146aa generated from circSHPRH exhibited tumor specificity, and this encoded peptide was abundantly expressed in the normal human brain and downregulated in 81% of glioblastomas. SHPRH-146aa was able to protect full-length SHPRH from DTL-induced ubiquitination. As a key tumor suppressor, SHPRH contributes to the inhibition of tumorigenesis and progression (24). CircRHOT1 was highly expressed in advanced hepatocellular carcinoma (HCC) tissues and inhibited HCC progression by recruiting TIP60 to initiate NR2F6 transcription. It was strongly associated with the prognosis of HCC (25). Currently, there are two main known modes of translation initiation for circRNAs, the internal ribosome entry site (IRES) and the N6-methyladenosine (m6A). m6A modification is one of the most common types of RNA modification in eukaryotes. It is widely involved in the regulation of biogenesis, splicing, translation, stability, and degradation of RNA. A recent study reviewed the functional crosstalk between m6A and circRNAs in cancer (26). Another mechanism has been proposed to activate the cap-independent pathway through the IRES, which is located in the 5′ UTR of mRNA. IRES initiates translation by directly recruiting ribosomes, an IRES-driven mechanism that has been explored in recent studies. Fan et al. (27) showed that certain short elements other than known IRES are sufficient to initiate circRNA translation. CircRNA-translated proteins also have certain biological roles. For example, during myogenesis, circZNF609 can undergo splice-dependent and cap non-dependent translation into proteins that specifically control myoblast proliferation (28).

In addition, the identification of differences in gene expression levels between cancer and control samples is a critical component of cancer biology research. Analyses involving multiple tumor cells and corresponding normal cells have found aberrant expression levels of circRNAs in different malignancies. A growing number of studies have shown that circRNAs are closely associated with the development of cancer and can be used as biomarkers (29–32). The main distinct advantage is their ability to be detected via RT-PCR of samples, as well as their great circulatory stability. Abnormal expression of circRNAs is correlated with tumor size, TNM stage, lymph node metastasis (LNM), and poor overall survival in TC (33). However, the relationship between PTC and circRNAs is still poorly understood. Those potential new roles of circRNAs need to be thoroughly investigated further.

## 3 CircRNAs in PTC

**Figure 1:**
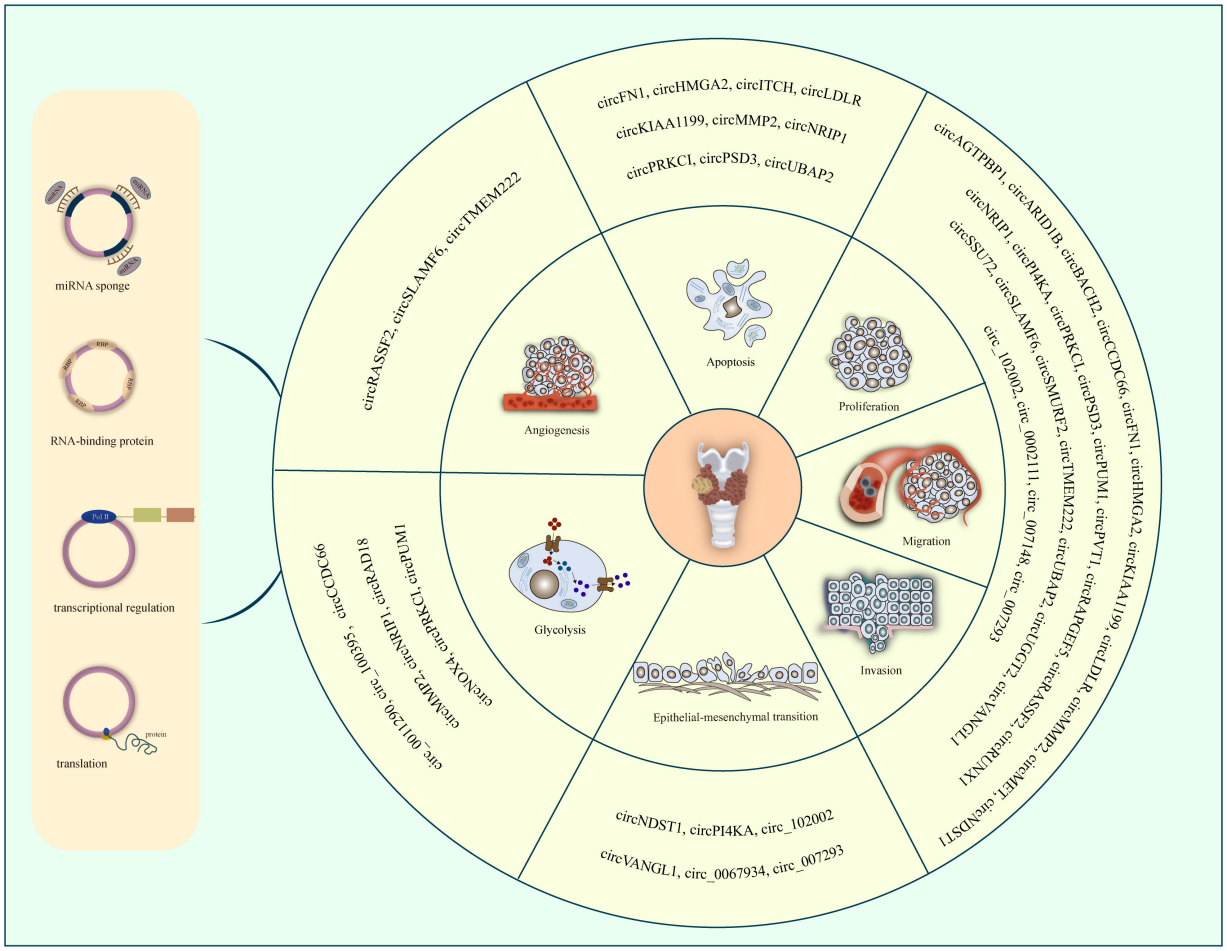
The regulatory uniqueness of circRNAs in PTC progression.

To date, a number of circRNAs have been found to be aberrantly expressed in PTC. They can regulate the progression of PTC through various aspects. However, the precise mechanisms by which circRNAs influence PTC development and progression remain unclear. Therefore, we summarize the dysregulated circRNAs in PTC reported in recent studies (**Table 1**). We can see most of the circRNAs are up-regulated in PTC. Moreover, they could act as miRNA sponges to carry out their functions. Some circRNAs have one or more miRNA binding sites that enable them to sequester miRNAs and further regulate the expression of their downstream target genes. illustrates the function of circRNAs in PTC. Further, we generalize the regulatory uniqueness of circRNAs in PTC in **Figure 1**.

Table 1Dysregulated circRNAs in PTC.

**Table:** 

CircRNAs up-regulated (↑)	miRNA	Target gene/ Pathway	Biological Function	Reference
circFN1 _055 (hsa_circ_0058124)	↓ miR-218-5p ↓ miR-940	↑ NOTCH3/GATAD2A	promotes cell proliferation, cell invasion, metastasis, and tumorigenicity	(34)
circFN1 _055 (hsa_circ_0058124)	↓ miR-370-3p	↑ LMO4	promotes cell viability, colony formation, migration, invasion and suppresses cell apoptosis	(35)
circRUNX1_005 (hsa_circ_0002360)	↓ miR-296-3p	↑ DDHD2	promotes cell proliferation, migration, and invasion	(36)
circARID1B_031 (hsa_circ_0001658)	↓ miR-671-5p	↑ PI3K/AKT/ITGA2	promotes cell proliferation and migration	(37)
circPVT1	↓ miR-195	↑ Wnt/β-catenin/VEGFA	promotes cell proliferation, migration, and invasion	(38)
circPVT1	↓ miR-126	↑ Bax/Bcl-2/PCNA	promotes cell viability, migration and invasion	(39)
circMMP2_005 (hsa_circ_0039411)	↓ miR-1179 ↓ miR-1205	↑ ABCA9 ↑MTA1	promotes cell growth, migration, invasion and suppresses cell apoptosis	(40)
circMMP2_005 (hsa_circ_0039411)	↓ miR-423-5p	↑ SOX4	promotes cell growth, migration, invasion, and glycolysis	(41)
circPSD3_017 (hsa_circ_0004458)	↓ miR-885-5p	↑ RAC1	promotes cell proliferation, suppresses cell cycle arrest and apoptosis	(42)
circLDLR_027 (hsa_circ_0003892)	↓ miR-195-5p	↑ LIPH	promotes cell proliferation, colony formation, migration, invasion and suppresses cell apoptosis	(43)
circLDLR _027 (hsa_circ_0003892)	↓ miR-637	↑ LMO4	promotes cell growth, migration, invasion and suppresses cell apoptosis	(44)
circLDLR_027 (hsa_circ_0003892)	↓ miR-326	↑ LASP1	promotes cell proliferation, migration, and invasion	(45)
circPRKCI _020 (hsa_circ_0067934)	↓ miR-1304	↑ CXCR1	promotes cell proliferation, migration, invasion and suppresses cell apoptosis	(46)
circPRKCI _020 (hsa_circ_0067934)	↓ miR-545-3p	↑ SLC7A11	promotes cell proliferation and suppresses cell apoptosis.	(47)
circPRKCI _020 (hsa_circ_0067934)	↓ miR-1301-3p	↑ HMGB1	promotes cell proliferation, migration, invasion and EMT	(48)
circNRIP1	↓ miR-195-5p	↑ P38 MAPK/JAK/STAT	promotes cell proliferation, invasion, and suppresses apoptosis	(49)
circNRIP1	↓ miR-653-5p	↑ PBX3	promotes cell proliferation, migration, invasion and suppresses cell apoptosis	(50)
circNRIP1	↓ miR-541-5p ↓ miR-3064-5p	↑ PKM2	promotes cell proliferation and glycolysis	(51)
circKIAA1199_022 (hsa_circ_0000644)	↓ miR-1205	↑ E2F3	promotes cell growth, migration, invasion and suppresses cell apoptosis	(52)
circNDST1 _011 (hsa_circ_0006943)	–	↑ PI3K/AKT/EMT	promotes cell proliferation, migration, invasion, and EMT	(53)
circSSU72_007 (hsa_circ_0009294)	↓ miR-451a	↑ AKT/S1PR2	promotes cell proliferation, migration, and invasion	(54)
circRAPGEF5_001 (hsa_circ_0079558)	↓ miR-26b-5p	↑ MET/AKT	promotes cell proliferation, motility and suppresses cell apoptosis	(55)
circUBAP2_046 (hsa_circ_0003141)	↓ miR-370-3p	↑ PI3K/AKT/Wnt	promotes cell proliferation, invasion and suppresses cell apoptosis	(56)
circSMURF2 (hsa_circ_102171)	–	↑ Wnt/β-catenin/CTNNBIP1	promotes PTC progression by activating Wnt/β-catenin pathway in a CTNNBIP1-dependent way	(57)
circUGGT2_065 (hsa_circ_0008274)	–	↑ mTOR/AMPK	promotes cell proliferation and invasion	(58)
hsa_circ_102002	↓ miR-488-3p	↑ HAS2	promotes cell proliferation, migration, and EMT	(59)
circPI4KA_028 (hsa_circ_0062389)	↓ miR-1179	↑ HMGB1	promotes cell proliferation, migration, and EMT	(60)
circPUM1	↓ miR-21-5p	↑ MAPK1	promotes cell proliferation, metastasis and glycolysis	(61)
circNOX4 _002 (hsa_circ_0023990)	↓ miR-485-5p	↑ FOXM1	promotes cell proliferation and glycolysis	(62)
hsa_circ_000121	↓ miR-4763 ↓ miR-6775	↑ SRC ↑ MMP-14	promotes the aggressiveness and lymph node metastasis	(63)
circBACH2_003 (hsa_circ_0001627)	↓ miR-139-5p	↑ LMO4	promotes cell proliferation, migration and invasion	(64)
circMET_004 (hsa_circ_0082003)	–	–	promotes cell proliferation, migration and invasion	(65)
circHMGA2_001 (hsa_circ_0027446)	↓ miR-129–5p	↑ CLDN1	promotes cell proliferation, migration, invasion and suppresses cell apoptosis	(66)
hsa_circ_0002111	–	–	promotes cell proliferation and invasion	(67)
hsa_circ_007148	–	–	associated with LNM	(68)
hsa_circ_007293	↓ miR-653-5p	↑ PAX6	promotes cell proliferation, invasion, migration, and EMT	(69)
circUGGT2_065 (hsa_circ_0008274)	↓ miR-154-3p	↑ SLC7A11	promotes cell migration and adhesion	(70)
circCCDC66	↓ miR-129-5p	↑ LARP1	promotes cell proliferation, invasion, and migration	(71)
circVANGL1	↓ miR-194	↑ ZEB1	promotes cell proliferation, invasion, migration, and EMT	(72)
circTMEM222_001 (hsa_circ_0011058)	↓ miR-335-5p	↑ YAP1	promotes cell proliferation, angiogenesis and radioresistance	(73)
circPSD3	↓ miR-7-5p	↑ METTL7B	promotes cell proliferation and invasion	(74)
circPRKCI_017 (hsa_circ_0122683)	↓ miR-335	↑ E2F3	promotes cell proliferation, invasion, and glycolysis	(75)
circRASSF2_009 (hsa_circ_0059354)	↓ miR-766-3p	↑ ARFGEF1	promotes cell proliferation, migration, invasion and angiogenesis	(76)
circSLAMF6_001 (hsa_circ_0000144)	↓ miR-1178-3p	↑ YWHAH	promotes cell proliferation, migration, invasion and angiogenesis	(77)
circFAM120B_004 (hsa_circ_0001666)	↓ miR-330-5p ↓ miR-193a-5p ↓ miR-326	↑ ETV4	promotes cell proliferation and arrest of cell cycle	(78)
circAGTPBP1_006 (hsa_circ_0087391)	↓ miR-34a-5p	↑ NOTCH1	promotes cell proliferation, migration, invasion, and metastasis	(79)

**Table:** 

CircRNAs down-regulated (↓)	miRNA	Target gene/ Pathway	Biological Function	Reference
hsa_circ_047771	↑ miR-522-3p	–	associated with BRAFV600 mutation, LNM, and advanced TNM stage	(68)
circITCH	↑ miR-22-3p	↓ CBL/β-catenin	suppresses cell proliferation and invasion and promotes cell apoptosis	(80)
circFAM53B (hsa_circ_0000266)	↑ miR-183-5p	↓ CCDC6	suppresses cell proliferation, migration, and invasion	(81)
hsa_circ_100395	–	↓ PI3K/AKT/mTOR	suppresses cell migration, invasion, glycolysis and downregulated PI3K/AKT/mTOR pathway	(82)

### 3.1 CircRNAs act as ceRNAs involved in PTC biological processes

Current research has found that circRNAs could act as ceRNAs to absorb miRNAs and participate in the regulation of PTC. CeRNA was first proposed by Pandolfi’s team in 2011 (83). They found that ceRNA molecules can compete to bind the same miRNAs through miRNA response elements (MREs) to regulate each other’s expression levels. It is known that miRNAs can cause gene silencing by binding to the 3’-UTR matched by mRNAs through the seed region, while ceRNA can regulate gene expression by competitively binding miRNAs.

According to multiple previous studies, some circRNAs could participate in one or more miRNA sponge pathways. For example, CircFN1 (hsa_circ_0058124) acts as an oncogenic driver that promotes PTC cell proliferation, tumor invasion, and metastasis. It could bind miR-218-5p to modulate the expression of GATAD2A, which plays an important role in transcriptional regulation. Likewise, it can bind to miR-940, which regulates the expression of MAPK1, an essential MAP kinase that integrates various biochemical signals to regulate cellular processes such as proliferation, differentiation, and transcriptional regulation (34). Moreover, circFN1 interacts with miR-370-3p to modify the expression of LMO4, which has the potential to function as an oncogene or a transcriptional regulator (35). Additionally, circFN1 promotes the progression of PTC by sponging miR-873-5p while restraining the malignancy of PTC cells by binding to FSTL1 (84).

Chu et al. (36) found that circRUNX1_005 (hsa_circ_0002360) promoted DDHD2 expression by sponging miR-296-3p. This gene encodes the protein phospholipase DDHD2, which is involved in membrane transport between the endoplasmic reticulum and the Golgi apparatus, thereby enhancing the proliferation, migration, and invasion of PTC cells. Similarly, silencing of circPSD3 (hsa_circ_0004458) significantly downregulated RAC1 expression by sponging miR-885-5p. It produces small plasma membrane-associated GTPases that bind to a variety of effector proteins to regulate cellular responses, thereby promoting PTC cell cycle arrest and apoptosis (42).

CircPVT1 has been reported to promote the expression of VEGFA to induce endothelial cell proliferation, promoting cell migration, inhibiting apoptosis, and inducing vascular permeabilization by sponging miR-195 (38), or by evaluating Bax/Bcl-2/PCNA to regulate apoptosis or proliferation of cells by sponging miR-126 (39). Similarly, circMMP2 (hsa_circ_0039411) (40, 41) regulates SOX4 expression by sponging miR-423-5p and translating a transcription factor protein. CircMMP2 also sponges miR-1179 to regulate the expression of ABCA9, transporting and translocating various molecules through extracellular and intracellular membranes. Moreover, it can bind miR-1205 to regulate the expression of MTA1, which is involved in transcription, migration, invasion, and glycolysis of PTC cells.

According to recent research, circLDLR (hsa_circ_0003892) functions as a sponge for miR-195-5p to regulate the catalytic lipid-mediated enzyme, lipase H (LIPH) (43). Moreover, circLDLR interacts with miR-637 to impact the expression of LMO4 and also interacts with miR-326 to regulate the expression of LASP1. LASP1 encodes for an actin-dependent signaling protein that binds to the actin cytoskeleton at the cell membrane’s extension to promote cell viability, migration, and invasion (44, 45). A novel oncogenic RNA, circBACH2, could be involved in the progression of PTC by acting as miR-139-5p sponge and then relieving suppression of the target LMO4 (64).

Furthermore, circPRKCI (hsa_circ_0067934) has been found to serve as a sponge for miR-1304 to modulate the expression of CXCR1, which encodes the receptor for IL8 (46). In addition, it interacts with miR-545-3p to modify the expression of SLC7A11 (47). This encoded protein has been recognized as the primary mediator of Kaposi’s sarcoma-associated herpes and is allowed to follow fusion and entry into cells. Also, circPRKCI acts as a sponge for miR-1301-3p to modulate the expression of HMGB1, which encodes a non-histone nuclear DNA-binding protein that regulates transcription and DNA organization (48). This protein is involved in various cellular processes, such as inflammation, cell differentiation, and cell migration.

Recently, studies reported that circNRIP1 could bind miR-195-5p and miR-653-5p to regulate the expression of PBX3, a transcription regulator critical for animal organ and neuronal development, as well as regulation by RNA polymerase II (49, 50). Additionally, circNRIP1 can sponge miR-541-5p, playing a role in the regulation of PKM2, a crucial glycolytic enzyme (51).

Another hotly studied circRNA, circKIAA1199 (hsa_circ_0000644) can sponge miR-1205, which regulates the expression of E2F3 (52). Notably, E2F3 is involved in the regulation of genes essential for cell cycle progression and is a crucial oncogene in several tumors. Also, circKIAA1199 has been reported to function as a sponge for miR-671-5p, which leads to the regulation of ANXA2 expression (85). ANXA2 is a member of the membrane-linked protein family and acts as an autocrine factor, promoting osteoclast formation and bone resorption.

Overall, increasing efforts have been made to explore the mechanisms by which circRNAs regulate PTC progression. Such findings provide new insights into the physiological and pathological processes of PTC.

### 3.2 CircRNAs involved in associated signaling pathways in PTC

**Figure 2:**
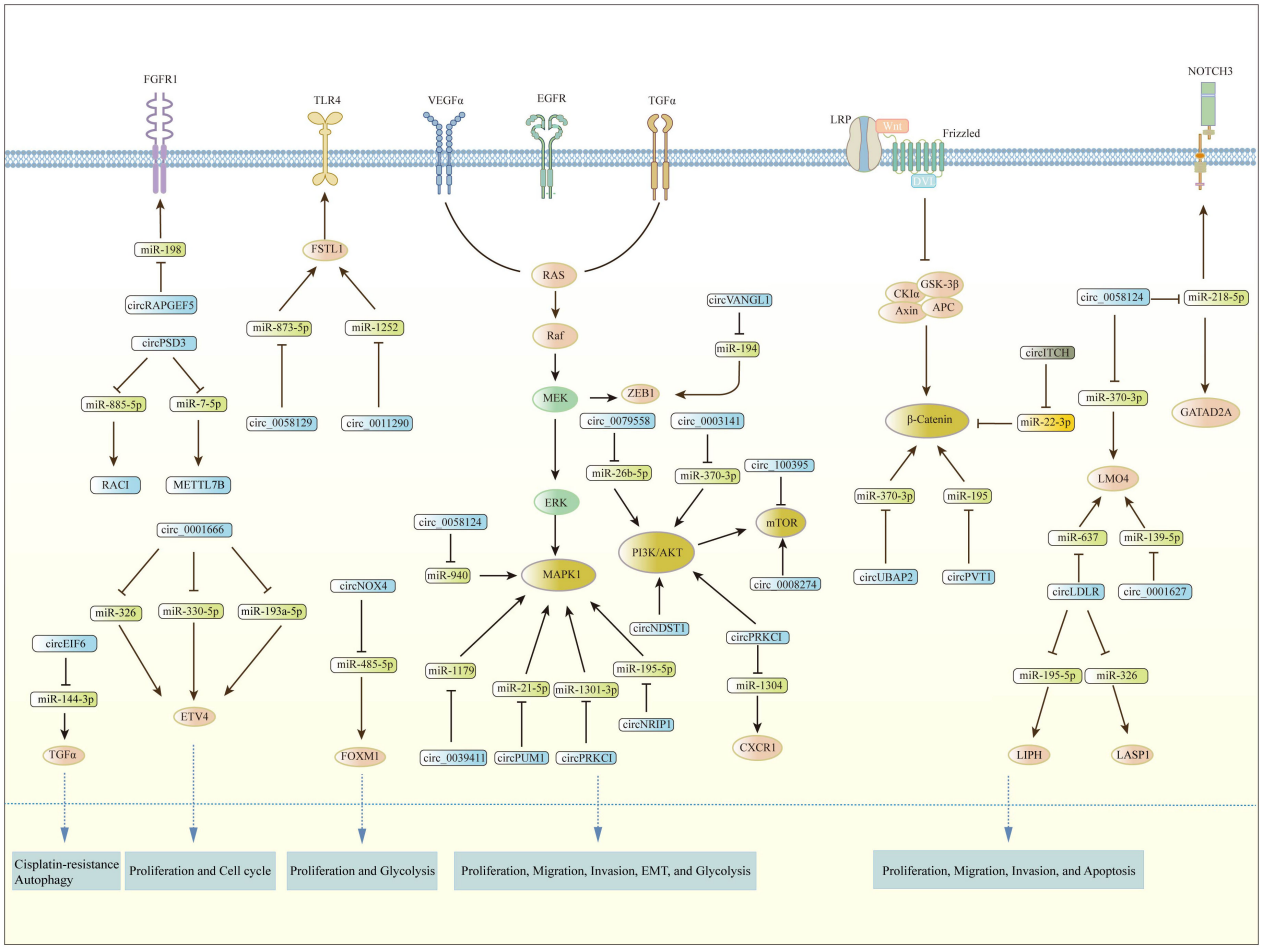
The involvement of circRNAs in relevant signaling pathways in PTC.

CircRNAs have been shown to function as tumor suppressors or oncogenes by regulating gene expression and modulating signaling pathways. Multiple signaling pathways are involved in the development and progression of PTC, including PI3K/AKT, Wnt/β-catenin, MAPK/ERK, AMPK/mTOR, and others. **Figure 2** demonstrates the involvement of circRNAs in relevant signaling pathways in PTC. These pathways are frequently dysregulated in PTC and play critical roles in regulating cell proliferation, migration, invasion, metastasis, apoptosis, angiogenesis, glycolysis, and EMT.

#### 3.2.1 PI3K/AKT signaling pathway

The PI3K/AKT pathway is frequently activated in various human cancers, making it an attractive therapeutic target (86). It is frequently activated by mutations in genes such as PIK3CA, PTEN, and AKT. In PTC, activation of the PI3K/AKT pathway increases cell survival and resistance to apoptosis, as well as angiogenesis and migration. PI3Ks are divided into three classes and contain many lipid kinase enzymes. Receptor tyrosine kinases (RTKs), G protein-coupled receptors (GPCRs), RAS, and Rho-GTPases all have the ability to activate Class IA PI3Ks. The downstream signaling events mediated by PIP3 result in cell growth, proliferation, autophagy, and apoptosis (87).

Several circRNAs have been found that regulate the PI3K/AKT pathway and induce PTC progression. For example, circNDST1 was reported to be significantly up-regulated in PTC. After knocking down circNDST1, the proliferation, migration, and invasion abilities of PTC cells were obviously suppressed. Meanwhile, it inhibited the activation of the PI3K/AKT pathway (53). The circSSU72/miR-451a/S1PR2 axis could also regulate PTC progression by the AKT pathway, which could serve as a novel therapeutic target in PTC (54). Moreover, circRAPGEF5 (hsa_circ_0079558) could activate the MET/AKT pathway via miR-26b-5p, which subsequently promoted the progression of PTC (55). CircUBAP2 (hsa_circ_0003141), an oncogenic molecule, is highly expressed in PTC tissues and cells. It may regulate PTC development through the PI3K/Akt pathway (56). Similarly, circ_0067934 was highly expressed in PTC. It was found to promote EMT and activate the PI3K/AKT pathway to facilitate the malignant behavior of PTC (88). In contrast, circ_100395 may play an anti-oncogenic role in PTC cells through the inhibition of the PI3K/AKT/mTOR pathway (82).

#### 3.2.2 Wnt/β-catenin signaling pathway

The Wnt**/**β-catenin signaling pathway is a highly conserved signaling cascade that regulates cell proliferation, differentiation, and tissue homeostasis during embryonic development and in tissues. The pathway is activated by the binding of Wnt ligands to Frizzled (FZD) receptors and low-density lipoprotein receptor-related protein (LRP) co-receptors on the cell surface. It is transduced in cells via the β-catenin-dependent/canonical or β-catenin-independent/non-canonical pathways. It was reported that the abnormal activation of the Wnt**/**β-catenin signaling pathway is closely related to the progression of PTC. Moreover, dysregulation of the Wnt pathway is associated with the loss of tumor suppressor genes, such as APC and AXIN, and the activation of oncogenes, such as β-catenin and cyclin D1.

There are several circRNAs that have been reported to regulate the Wnt pathway in PTC (89). For instance, Chen et al. found that circNEK6 (hsa_circ_0088483) affects FDZ8 expression via the Wnt pathway. Its overexpression successfully enhanced the proliferation and invasion of PTC cells (90). Besides, it was reported that the Wnt/β-catenin signaling pathway was also significantly promoted by the overexpression of circPVT1 (38). Additionally, miR-370-3p was reported to regulate the Wnt pathway in a variety of cancers. Hsa_circ_0003141 was reported to regulate the Wnt pathway by miR-370-3p to play its role in PTC. Knockdown of hsa_circ_0003141 inhibited cell proliferation and invasion, while induced cell apoptosis of PTC (56). CTNNBIP1 is a β-catenin-interacting protein. CircRNA_102171 could exert a pro-carcinogenic role in PTC. It was found that circRNA_102171 could bind to CTNNBIP1, which in turn promoted the formation of the β-catenin/TCF complex and activated the Wnt/β-catenin pathway (57). Furthermore, FZD8 is reported as a Wnt receptor in the canonical Wnt signaling pathway that has been identified as a therapeutic target for cancer (91). MiR-345-5p, which was sponged by circPRS28 (hsa_circ_0049055), was a direct regulator of the functional gene FZD8 during PTC cell development (92). The relationship between circRPS28/miR-345-5p/FZD8 and the Wnt signaling pathway is left unexplored.

#### 3.2.3 MAPK/ERK and AMPK signaling pathway

The MAPK pathway is a signal-transduction system. Its constitutive activation is crucial for the growth of PTC. Through critical proteins, such as receptor tyrosine kinases, MAPKs control crucial physiological processes involved in cell proliferation, differentiation, and development. Growth factor binding to a receptor tyrosine kinase receptor triggers the activation of downstream pathways. For example, Li C et al. (49) reported that up-regulation of circNRIP1 inverted the inhibitive function of miR-195-5p on the P38 MAPK pathway. The AMP-activated protein kinase (AMPK) is an energy-sensing protein that regulates cellular metabolism and maintains energy homeostasis. AMPK acts as a potential tumor suppressor since it is located at the entrance of a tumor suppression network that regulates cell growth and proliferation in stress responses. The AMPK pathway is activated by various stimuli that increase the cellular AMP/ATP ratio, such as nutrient deprivation, exercise, and hypoxia. It plays an important role in regulating tumor growth and metabolism, including in PTC. Activating AMPK may promote tumor survival and growth by maintaining AMPK activity and the ability to adapt to metabolic stress (93). CircUGGT2 (has_circ_0008274) deletion was found to impair the AMPK pathway by decreasing AMPK phosphorylation (p-AMPK) and promoting the mTOR pathway by increasing mTOR phosphorylation (p-mTOR) in TPC-1 cells. CircUGGT2 attenuation inhibited the AMPK/mTOR signaling pathway (58). Furthermore, studies have shown that AMPK activation can enhance the sensitivity of TC cells to chemotherapeutic agents, such as doxorubicin and cisplatin, suggesting that AMPK activation may be a promising therapeutic strategy for treatment.

#### 3.2.4 Other signaling pathways

The NOTCH signaling pathway regulates cell differentiation and has been linked to several types of cancer, including PTC. It is frequently activated by mutations in genes, such as NOTCH1 and JAG1. The function of NOTCH1 in tumorigenesis has been extensively studied. *In vitro* studies have confirmed that overexpression of NOTCH1 was associated with resistance to cancer therapy. Research has identified that circAGTPBP1, as an oncogene, could regulate the NOTCH pathway through the miR-34a-5p/NOTCH1 axis and promote the progression of PTC (79). Besides, circFN1 was reported to promote PTC tumorigenesis and invasiveness through the NOTCH3 pathway. The knockdown of circFN1 significantly decreased cell viability, migration, and invasion. Furthermore, silencing circFN1 significantly suppressed cell migration (34). CircNRIP1 was found to modulate the JAK/STAT pathways and affect the cell functions and growth of xenografts (49).

In summary, the regulation of related signaling pathways plays a critical role in PTC progression. Those findings go far toward the progression of a novel therapy for PTC. However, the molecular mechanisms underlying the circRNAs’ regulation of those pathways in PTC are largely unknown. The exploration of circRNAs may provide new insights into PTC pathogenesis, and targeting those pathways may be a promising therapeutic approach for treatment.

### 3.3 Role of circRNAs in EMT in PTC

Epithelial-mesenchymal transition (EMT) is a process in which epithelial cells acquire mesenchymal features. EMT confers metastatic properties to cancer cells by enhancing cell migration and invasion, which is considered a marker of carcinogenesis (94, 95). In recent years, a growing number of studies have focused on the role of circRNAs in EMT in PTC. A study reported that circNDST1 (hsa_circ_0006943) overexpression boosted PTC progression through the activation of EMT in a CSNK2A1-dependent manner (53). Circ_102002 was found to facilitate metastasis of PTC by regulating the miR-488-3p/HAS2 axis. Inhibition of circ_102002 downregulated HAS2 levels, suppressed the phosphorylation of FAK and AKT, regulated expressions of E-cadherin and N-cadherin, and inhibited PTC metastasis (59). CircPI4KA (hsa_circ_0062389) is also involved in EMT via sponging miR-1179 and thus regulates HMGB1 expression. They found that circPI4KA depletion significantly increased E-cadherin and decreased N-cadherin (60). Moreover, silencing of circ_007293 inhibited EMT, as indicated by suppressed N-cadherin and vimentin expressions and increased E-cadherin expression (69). Besides, circPRKCI (hsa_circ_0067934) had a similar effect on promoting PTC progression by regulating EMT (88). Overall, these important features in EMT confirm the potential role of circRNA in PTC therapy.

### 3.4 Role of circRNAs in glycolysis in PTC

A hallmark of cancer cells is a change in energy metabolism, characterized by the preferential use of glycolysis for energy production. Although not as efficient as oxidative phosphorylation in terms of net ATP production, cancer cells adapt by increasing glucose uptake and promoting the rate of glycolysis. Moreover, intermediates of glycolytic metabolism play a key role in macromolecular biosynthesis. Targeting glycolysis remains an attractive intervention, and recent preclinical studies support its efficacy. Several studies have explored potential therapeutic opportunities for the cancer-specific effects of glycolysis inhibitors (96).

There are several circRNAs that are involved in the glycolytic process in PTC. CircNRIP1 enhances glycolysis in PTC cells by upregulating PKM2 levels and sponging miR-541-3p and miR-3064-5p (51). Li Y et al. (61) showed that knockdown of circPUM1 impedes cell growth, metastasis, and glycolysis of PTC via enhancing MAPK1 expression by sponging miR-21-5p. Downregulation of circPUM1 resulted in decreased expression of hexokinase 2 (HK2) and blocked glycolysis in PTC. Hsa_circ_0023990 promotes tumor growth and glycolysis in dedifferentiated TC via positively regulating the miR-485-5p/FOXM1 axis (62). Data demonstrated that glucose uptake, lactate production, and glycolytic genes (GLUT1, HK2, and LDHA) were all inhibited by circ_100395 overexpression (82). Hsa_circ_0011290 regulates glycolysis by regulating the miR-1252/FSTL1 axis. Meanwhile, glucose metabolism was significantly switched with decreased glucose uptake and lactate production (97). Also, it is found that circCCDC66 promotes the proliferation and migration of PTC cells by regulating miR-211-5p/PDK4, which in turn regulates glucose metabolism (98). CircRAD18 is involved in reprogramming glucose metabolism, and silencing of circRAD18 significantly inhibits cellular glucose uptake and lactate production in PTC cells by regulating miR-5166/PDK1 (99).

## 4 Clinical implications of circRNAs in PTC

Likewise, circRNAs have potential value as biomarkers in diagnosis, prognosis, and even treatment. Due to the lack of specific symptoms, PTC is often difficult to diagnose at an early stage. Early identification of the advanced PTC among many thyroid cancers is an urgent clinical issue to be addressed. A number of circRNAs have tissue-specific and developmental stage-specific expression patterns.

### 4.1 Role of circRNAs as diagnostic biomarkers

Most circRNAs have been found to be upregulated in PTC tissues or cells compared to normal. Bai C et al. (63) found that hsa_circ_000121 had good sensitivity and specificity for diagnosing PTC lymph node metastasis, with a cut-off value of 0.796. Cai X et al. (64) found that circBACH2 had a good diagnostic value for PTC with an AUC of 0.8631. In addition, hsa_circ_0082003 has potential as a biomarker for the diagnosis of PTC (65), as well as hsa_circ_0027446 (66). CircPSD3 (hsa_circ_0002111) was suggested as a potential diagnostic tumor marker for PTC, with an AUC of 0.833 (95% CI = 0.77-0.89, p < 0.01) (67). Similarly, circMAN1A2 has an AUC of 0.734 (100), hsa_circ_047771 has an AUC of 0.876 (95% CI = 0.78-0.94), and hsa_circ_007148 has an AUC of 0.846 (95% CI =0.75-0.96) (68). Studies have shown that exosomal circ_007293 regulated PTC cell invasion, migration, proliferation, and EMT, as well as induced PAX6 expression by sponging miR-653-5p (69). Further studies found that three differentially regulated circRNAs, including has_circ_007293, has_circ_031752, and has_circ_020135, were upregulated and confirmed in the serum of PTC patients (101). Additionally, these findings suggest that exosome circRNAs might be potential diagnostic molecular biomarkers for PTC.

### 4.2 Role of circRNAs as prognostic biomarkers

Recently, several studies have found that dysregulation of circRNAs is associated with a poor prognosis in PTC, including tumor size, LNM, TNM stage, and even postoperative recurrence. The correlation between circRNAs and clinical features of PTC is shown in **Table 2**.

**Table 2:** The correlation between circRNAs and clinical features of PTC.

Clinical features	Upregulated circRNAs	Downregulated circRNAs
Tumor size >3 cm	circLDLR (44), circPSD3 (74), circFOXM1 (102)	
Tumor size ≥2 cm	hsa_circ_0079558 (55), hsa_circ_0008274 (70)	
Tumor size ≥1cm	circPVT1 (38), circNRIP1 (50), hsa_circ_0000644 (52), hsa_circ_0079558 (55), hsa_circ_0003141 (56), circPUM1 (61), hsa_circ_0082003 (65), hsa_circ_0002111 (67), hsa_circ_0008274 (70), circCCDC66 (71), circVANGL1 (72), hsa_circ_0122683 (75), hsa_circ_0000144 (77), circAGTPBP1 (79), hsa_circ_0000266 (81)	
LNM	circLDLR (44), hsa_circ_0079558 (55), circBACH2 (64), hsa_circ_007293 (69), hsa_circ_0008274 (70), hsa_circ_0011058 (73), circPSD3 (74), hsa_circ_0001666 (78), circAGTPBP1 (79)	
TNM stage	circPVT1 (38), circLDLR (44), hsa_circ_0067934 (48), circNRIP1 (50), hsa_circ_0079558 (55), hsa_circ_0003141 (56), circPUM1 (61), circBACH2 (64), hsa_circ_0082003 (65), hsa_circ_0002111 (67), hsa_circ_007293 (69), hsa_circ_0008274 (70), circCCDC66 (71), circVANGL1 (72), hsa_circ_0011058 (73), circPSD3 (74), hsa_circ_0122683 (75), hsa_circ_0059354 (76), hsa_circ_0000144 (77), circAGTPBP1 (79)	hsa_circ_0000266 (81)

In addition, data obtained by COX regression analysis also supports the potential value of circRNAs in terms of prognosis. For example, it was found that patients with high expression of has_circ_0067934 showed a lower survival period. Cox model analysis indicated that hsa_circ_0067934 was an independent risk factor for prognosis (RR = 4.385, 95% CI = 1.087-17.544, p = 0.038) (88). Several high expression levels of circRNAs were associated with a poor prognosis of PTC, such as hsa_circ_102002 (59), circPUM1 (61), circBACH2 (64), hsa_circ_0027446 (66), hsa_circ_0008274 (70), circCCDC66 (71), and hsa_circ_0011058 (73). In contrast, low circ_100395 expression was linked to a poor prognosis in PTC (82). However, low hsa_circ_047771 expression was associated with the BRAF^V600^ mutation, LNM, and TNM stage (p < 0.05). Furthermore, a high expression of hsa_circ_007148 was significantly correlated with LNM (P < 0.05) (68).

### 4.3 Role of circRNAs in treatment

CircRNAs have garnered growing interest in tumor research and hold promise for intervention or regulatory therapy. Drug resistance is a key factor affecting cancer outcomes. Several studies have demonstrated the association of circRNAs with chemoresistance in cancer treatment. For instance, the circEIF6/miR-144-3p/TGF-α pathway was found to be associated with reduced sensitivity of ATC to cisplatin resistance. CircEIF6 could promote tumor growth by regulating miR-144-3p/TGF-α, while knockdown of circEIF6 enhanced cisplatin sensitivity *in vivo*. This finding suggests a potential therapeutic target for overcoming cisplatin resistance in TC (103). However, the downstream signaling molecules of TGF-α need to be further investigated. Additionally, due to the undifferentiated phenotype of TC and its aggressive nature, resistance to conventional treatments such as radiotherapy and chemotherapy is frequently observed in ATC, including cisplatin resistance (104). In breast cancer, circCDYL2 was able to maintain downstream AKT and ERK1/2 activity, thus promoting trastuzumab resistance in HER2-positive breast cancer patients (105). Furthermore, circCPM promoted resistance to 5-FU in gastric cancer and modulated autophagy by targeting PRKAA2 (106). In endometrial cancer, resistance to paclitaxel was found to be mediated by the key oncogenic circ0007534 (107). These studies suggest that circRNA-targeted therapy may have a role in reversing cancer chemoresistance.

In addition, circRNAs demonstrate promise as a drug carrier for the treatment of cancer. They exhibit robust stability and resistance to degradation by nucleases. These characteristics enable prolonged circulation in the body. CircRNAs can achieve targeted therapy by interacting with specific miRNAs. In PTC, certain miRNAs may be dysregulated, contributing to cancer progression. CircRNAs can be engineered to bind to these miRNAs, thereby restoring their normal levels and inhibiting cancer cell growth and dissemination. Also, aberrant expression of specific key genes closely correlates with cancer development in TC. CircRNAs serve as drug carriers, delivering specific siRNA or gene sequences to target genes. Pisignano G et al. (108) summarized ongoing research on evaluating the potential therapeutic effects of circRNA application in clinical practice for cancer patients. A study attempted to develop therapeutic mesenchymal stem cells (MSCs) containing a suicide-inducing gene, and they demonstrated the effectiveness of this approach in ATC therapy (109). The immunogenicity of extracellular vesicles derived from MSC is relatively low, making them potential alternatives to cell therapy. A recent review highlights the therapeutic options and biological applications of MSCs-derived extracellular vesicles. They are capable of transmitting signals or delivering biological materials to diseased sites in the body and then regulating therapy (110). However, the research on utilizing MSCs as carriers to load circRNAs for the treatment of TC remains in its early stages. More intensive studies in aggressive TC are highly needed to provide novel approaches for tumor therapy.

## 5 Discussion

PTC is a multifactorial disease, the prevalence of which is increasing year by year, and its diagnosis, treatment, and prognosis are still controversial in clinical practice (111). A recent study on the Chinese population found a rapid increase in the incidence of TC and a modest increase in mortality from 2005 to 2015 (112). In addition, based on decades of global epidemiological data, the overall incidence of TC was found to have increased by 3% per year between 1974 and 2013, with a concomitant 1.1% per year increase in mortality based on incidence. For advanced PTC, the incidence and mortality rates are also increasing annually in most countries (113). PTC accounts for approximately 85% of TC and includes follicular, diffuse sclerosing, hypercellular, and columnar cell subtypes. Of these, the diffuse sclerosing subtype has a 100% LNM rate, often occurs in children and young adults, and shows a poor prognosis. Tumor diagnosis and treatment have now moved into the era of precision medicine, and several molecular markers such as BRAF, RAS, RET/PTC, and PAX8/PPARγ have been used to improve the accuracy and timeliness of thyroid nodule diagnosis, as well as to reach the molecular level of the subtype classification of PTC. Recently, patients with RET/NTRK fusion were found to have worse clinical outcomes than patients with BRAF-mutated disease (114). Therefore, the early identification of highly aggressive, poorly differentiated, and poorly prognosed TC is of great importance. We provided a detailed review of recent findings in the field of circRNAs for PTC. Focusing on the biological functions of circRNAs and their related signaling pathways in PTC, as well as discussing the role of circRNAs in the EMT and glycolysis processes. Then, we discussed the clinical implications of circRNAs in PTC and found that circRNAs may be a new therapeutic approach for the treatment of TC.

CircRNAs have been found to be widely expressed in various cancers and are associated with tumorigenesis and progression. Because of the specific features of circRNAs, such as good stability, abundance, sensitivity, and specificity, they are gradually being proposed as biomarkers. The detection of circRNA in serum and FNA samples as a non-invasive diagnostic tool for PTC has several advantages over traditional diagnostic methods. Firstly, circRNA has the potential to improve the accuracy of PTC diagnosis as it is specific to cancer cells and could be used to differentiate between benign and malignant tumors. Secondly, it is less invasive than a tissue biopsy, and samples are more readily available. Further, this modality has the potential to improve the management of PTC by providing an early and accurate diagnosis, which could contribute to timely and appropriate treatment. Current studies have provided promising and informative evidence that confirms the viability of circRNAs as biomarkers.

With increasing evidence revealing the role that circRNAs play in multiple signaling pathway processes, this provides more focus points for PTC treatment. Among the processes associated with PTC, one of great importance is EMT. Several studies have reported that circRNAs have a distinct, easily measured, and observed corresponding expression during EMT, which is thought to be of potential value in inhibiting the malignant progression of TC. In spite of these exciting advances, the research on circRNAs still faces a few limitations. Firstly, due to the different sequencing methods, data analysis pipelines, and detection tools, the current databases of circRNAs are not uniform and very complete. This makes it difficult to compare the results of different studies. Furthermore, the limited sample sizes of studies make it difficult to generalize the results to larger populations. Numerous circRNAs have been identified and found to have multiple functions through diverse mechanisms. This complexity makes it challenging to fully understand their role in PTC.

## 6 Future perspectives

In recent years, significant achievements have been made in the fields of circRNAs and PTC, yet the clinical application of circRNAs in PTC still faces numerous challenges. Firstly, existing studies are mainly focused on the PTC, while other subtypes of TC are insufficient, particularly MTC and ATC. Future research should allocate more attention to circRNAs in these subtypes. Secondly, due to the predominantly low expression levels of circRNAs, investigating efficient extraction and detection methods for circRNAs could enhance their utility as biomarkers. It is necessary to monitor relapse and progression using reliable biomarkers in long-term follow-up studies. Additionally, reliance on a single circRNA may not provide adequate support for cancer diagnosis or prognosis prediction. Hence, the utilization of a combination of cancer-related circRNA panels could prove valuable as biomarkers in the future. Moreover, despite the broad potential applications of circRNAs, current research predominantly focuses on their role as miRNA sponges, which is overly narrow. Other mechanisms of circRNAs warrant further investigation. Last but not least, an increasing number of studies are delving into the role of circRNAs in chemoresistance and cancer therapy. This represents a novel avenue for future investigation and merits thorough exploration. Nevertheless, additional research and validation are indispensable prior to the clinical implementation of circRNA-targeted treatments. Addressing concerns pertaining to safety, efficacy, drug release, and *in vivo* targeting is imperative.

In conclusion, circRNAs undoubtedly have enormous potential for cancer diagnosis and treatment. Through further research on circRNAs biogenesis and other mechanisms, alongside the development of detection technologies and comprehensive clinical trials, circRNAs will play a significant role in the diagnosis, monitoring, and treatment of PTC.

## Author contributions

JM: Conceptualization, Data curation, Formal Analysis, Funding acquisition, Investigation, Validation, Writing – original draft. JX: Data curation, Formal Analysis, Investigation, Writing – original draft. XZ: Data curation, Formal Analysis, Software, Validation, Visualization, Writing – original draft. JQ: Supervision, Validation, Visualization, Writing – review & editing, Funding acquisition.
